# AARS1‐mediated PHGDH lactylation promotes hepatocellular carcinoma progression by enhancing serine biosynthesis

**DOI:** 10.1002/ctm2.70899

**Published:** 2026-11-14

**Authors:** Qing Zhu, Nanyan Yang, Xiangzhuo Miao, Shiting Xu, Junming Chen, Huohui Ou, Jianmin Nie, Pengyu Zhu, Yinghao Fang, Dinghua Yang

**Affiliations:** ^1^ Unit of Hepatobiliary Surgery, Department of General Surgery Nanfang Hospital, Southern Medical University Guangzhou China; ^2^ Department of Thoracic Oncology and Surgery, China State Key Laboratory of Respiratory Disease & National Clinical Research Center for Respiratory Disease The First Affiliated Hospital of Guangzhou Medical University Guangzhou China; ^3^ State Key Laboratory of Respiratory Disease, Guangzhou Institute of Cancer Research, The Affiliated Cancer Hospital Guangzhou Medical University Guangzhou China

**Keywords:** alanyl‐tRNA synthetase 1, hepatocellular carcinoma, lysine lactylation, phosphoglycerate dehydrogenase, redox homeostasis, serine metabolism

## Abstract

**Background:**

The recently described lactyltransferase activity of alanyl‐tRNA synthetase 1 (AARS1) raises questions about its metabolic functions in hepatocellular carcinoma. We examined phosphoglycerate dehydrogenase (PHGDH) as a potential lactylation substrate linking AARS1 to serine metabolism and tumour growth.

**Methods:**

Public expression data and patient tissues were studied alongside proteomic, lactylomic and targeted metabolomic profiles. Cellular and xenograft experiments used gene depletion, overexpression and reconstitution with wild‐type or mutant proteins. Immunoprecipitation and activity measurements assessed PHGDH regulation. Survival was examined with Kaplan‒Meier estimates and Cox regression, while experimental groups were compared by *t*‐tests or analysis of variance.

**Results:**

Of 140 paired specimens, 119 tumours (85%) had higher AARS1 mRNA than their non‐tumour counterparts. Elevated AARS1 independently predicted poorer survival, and E2F4 depletion lowered AARS1 expression. AARS1 supported malignant phenotypes, lactate production and PHGDH lactylation. Mass spectrometry detected lactylation at PHGDH‐K394. Relative to wild‐type PHGDH, K394R showed less lactylation and supported lower lysate enzyme activity, serine, glycine and glutathione levels. The mutant also increased reactive oxygen species, provided weaker phenotypic rescue and supported less xenograft growth. Depleting AARS1 reduced enzyme activity and malignant phenotypes in the wild‐type background; no further significant reduction was detected with K394R. β‐Alanine inhibited xenograft growth, but PHGDH‐K394 alone could not account for its effects.

**Conclusions:**

AARS1‐associated PHGDH‐K394 lactylation may connect serine metabolism and redox regulation to hepatocellular carcinoma progression. Establishing therapeutic relevance will require site‐specific mechanistic experiments and additional preclinical validation.

## HIGHLIGHTS

- AARS1 is elevated in hepatocellular carcinoma and predicts poorer survival.
- AARS1 promotes lactate production and mediates PHGDH K394 lactylation in hepatocellular carcinoma.
- PHGDH K394 lactylation supports serine metabolism and redox balance.
- PHGDH K394R weakens tumor phenotypes and xenograft growth.

## Graphical abstract

AARS1 is upregulated in hepatocellular carcinoma (HCC) and is associated with PHGDH‐K394 lactylation, which enhances PHGDH activity, serine/glycine metabolism and redox homeostasis. This metabolic adaptation promotes HCC proliferation, migration/invasion and xenograft growth, as supported by clinical samples and wild‐type (WT)/K394R functional comparisons.

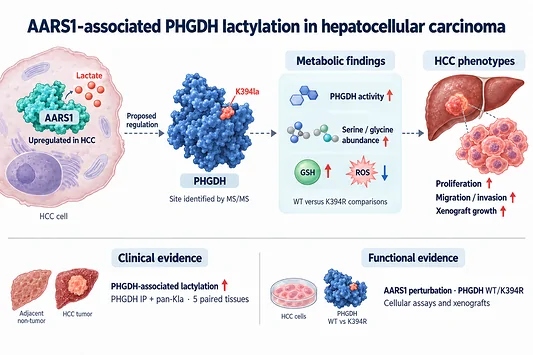

## 1 BACKGROUND

New therapeutic targets for hepatocellular carcinoma (HCC) require a clearer understanding of the processes that sustain its progression. HCC accounts for most primary liver cancers and contributes substantially to cancer mortality worldwide.^1^, ^2^

Cancer cells alter their metabolic programs to meet the demands of growth.^3^, ^4^ Increased glycolysis despite oxygen availability can raise lactate concentrations both inside tumours and in their surrounding microenvironment.^5^ Lactate also carries regulatory information: its signalling functions influence tumour behaviour, immune organization and responses to oxidative stress.^6^ One route through which lactate connects metabolism with gene expression and protein activity is lysine lactylation (Kla), a post‐translational modification derived from this metabolite.^7^ In HCC, evidence linking lactylation to disease progression has outpaced knowledge of the enzymes that install it, the substrates involved, and the resulting metabolic changes.^8^

In addition to its established contribution to translation, alanyl‐tRNA synthetase 1 (AARS1) can sense intracellular lactate and transfer lactyl groups to lysine residues.^9^, ^10^ Reports connecting this activity with proliferation, escape from immune surveillance, metabolic adaptation and treatment resistance suggest functions extending beyond protein synthesis.^11^, ^12^, ^13^, ^14^, ^15^, ^16^ AARS1 could therefore translate lactate availability into changes in proteins that support tumour growth. Whether metabolic enzymes are regulated through this mechanism during HCC progression has not been resolved.

The entry reaction into de novo serine synthesis is catalyzed by phosphoglycerate dehydrogenase (PHGDH), a rate‐limiting enzyme in a pathway that supplies biosynthetic substrates and supports redox balance.^17^, ^18^ Several cancers increase PHGDH expression, and tumours dependent on this enzyme are sensitive to its depletion or pharmacological inhibition.^19^, ^20^, ^21^ HCC presents a different observation: increased serine metabolism has been reported alongside lower PHGDH expression.^17^ This mismatch points to regulation beyond enzyme abundance. Phosphorylation, acetylation, methylation and ubiquitination are among the modifications capable of regulating PHGDH.^22^ It remains uncertain whether lactylation also modifies PHGDH in HCC and, if so, how it relates to enzyme activity, downstream metabolites and malignant behaviour.

Our study examined PHGDH‐K394 as a candidate link between AARS1, serine metabolism and HCC phenotypes. Clinical observations were integrated with proteomic and metabolic measurements, genetic interventions and xenograft studies to investigate PHGDH lactylation, enzyme activity and redox regulation.

## 2 METHODS

### 2.1 Public database analysis

The TCGA‐LIHC, GEO‐GSE14520 and International Cancer Genome Consortium (ICGC) cohorts provided the expression and survival data for evaluating AARS1 in HCC. Tumour‒normal expression differences were tested with the Wilcoxon rank‐sum procedure. For each cohort, an optimal expression threshold was determined separately and used to define low‐ and high‐AARS1 groups. Kaplan‒Meier estimates described overall survival, with group differences evaluated by log‐rank testing. These analyses used R, and the significance threshold was *p* < .05.

### 2.2 Tissue specimen collection

Clinical material came from patients undergoing radical HCC resection at Nanfang Hospital, Southern Medical University, from November 2012 to November 2024. Immediately after surgery, tumour and adjacent non‐tumour liver specimens were placed in liquid nitrogen or preserved in paraformaldehyde. Patients consented to collection under hospital ethics approval NFEC‐2024‐335. Survival information was obtained during scheduled outpatient visits after discharge. Recurrence assessment used postoperative serum alpha‐fetoprotein elevation together with imaging evidence of intrahepatic or extrahepatic space‐occupying lesions.

### 2.3 Cell culture

All six cell lines—Huh7, SK‐hep1, HepG2, LM3, Hep3B and SNU‐449—were supplied by the Type Culture Collection of the Chinese Academy of Sciences in Shanghai, China. Culture medium consisted of Dulbecco's modified Eagle's medium (DMEM; C11995500BT; Thermo Fisher Technology) and 10% foetal bovine serum (FBS) (A3160801; Pricella Biotechnology). Incubators were humidified and maintained at 37°C with 5% CO_2_.

### 2.4 RNA isolation and quantitative real‐time reverse‐transcription polymerase chain reaction

RNA preparations from clinical specimens and HCC cell lines used TRIzol (15596018; Thermo Fisher Scientific) following the supplier's protocol. HiScript IV RT SuperMix (R222‐01‐AB; Vazyme Biotech) converted the RNA to cDNA. Amplification and fluorescence acquisition were carried out on a StepOnePlus instrument (Applied Biosystems) with SybrGreen qPCR MasterMix (P2102; Dongsheng Biotech). The primer sequences are listed below—18S rRNA: 5′‐GTAACCCGTTGAACCCCATT‐3′ (forward), 5′‐CCA TCCAATCGGTAGTAGCG‐3′ (reverse); β‐actin: 5′‐TGG CACCCAGCACAATGAA‐3′ (forward), 5′‐CTAAGTCA TAGTCCGCCTAGAAGCA‐3′ (reverse); AARS1: 5′‐TCACCCAAGAGTTTGGCATTC‐3′ (forward), 5′‐CCTGGGAGGATTTTGGTGTCA‐3′ (reverse); E2F4: 5′‐CACCACCAAGTTCGTGTCCC‐3′ (forward), 5′‐GCGTACAGCTAGGGTGTCA‐3′ (reverse).

Expression was calculated by the 2^−^ΔΔCt approach. Normalization used β‐actin for cell‐derived RNA and 18S rRNA for RNA obtained from clinical specimens.

### 2.5 Immunohistochemistry and haematoxylin and eosin staining

Clinical tissues preserved in 4% formalin were processed into 4‐µm paraffin sections. Xylene and ethanol were used for deparaffinization, and haematoxylin and eosin (H&E) staining was performed. For immunohistochemistry (IHC), sodium citrate at pH 6.0 provided antigen retrieval. Sections then spent 1 h at room temperature in Tris‐buffered saline (TBS) supplemented with 1% bovine serum albumin (BSA) and  .5% Triton. Endogenous peroxidase was blocked by 15 min of exposure to 3% hydrogen peroxide/methanol at room temperature. Primary antibody incubation used either anti‐AARS1 (A15017; ABclonal) or anti‐Kla (PTM‐1401; PTM BIO), each diluted 1:1000, overnight at 4°C. Bound antibody was visualized with the streptavidin‒peroxidase system (sp‐9001; ZSGB‐BIO), following the kit protocol. Independent assessment of HCC staining used a quick score with two components. Intensity was graded as absent (0), weak (1), moderate (2), or strong (3); positive‐cell proportions were graded as 1 for 1%‒25%, 2 for 25%‒75%, and 3 for 75%‒100%. Multiplication of these component scores produced an immunoreactivity score between 0 and 9.

### 2.6 Western blotting

Collected cells and specimens received one rinse in ice‐cold phosphate‐buffered saline (PBS) before a 30‐min extraction on ice with p0013 lysis buffer (Beyotime), following the product protocol. Protein concentrations were determined with the BCA Protein Assay Kit (ab102536; Abcam). Samples in Laemmli buffer were heated for 10 min at 95°C, resolved by sodium dodecyl sulfate‒polyacrylamide gel electrophoresis (SDS‒PAGE), and transferred onto polyvinylidene fluoride (PVDF). Blocking used 5% nonfat milk in Tris‐buffered saline with Tween 20 (TBST) for 1 h at room temperature. Primary antibody binding proceeded at 4°C overnight; secondary IgG incubation lasted 1 h at room temperature. Chemiluminescence from an ECL substrate (Fdbio Science) was recorded on a FluorChem E instrument (ProteinSimple). The horseradish peroxidase (HRP)‐linked secondary reagents were anti‐rabbit A0208 and anti‐mouse A0216 (Beyotime). Primary reagents comprised anti‐AARS1 (A15017; ABclonal), anti‐Kla (PTM‐1401; PTM BIO), anti‐PHGDH (14719‐1‐AP; Proteintech), anti‐HA (AE036; ABclonal), anti‐FLAG (AE063; ABclonal), anti‐LDHA (21799‐1‐AP; Proteintech), anti‐PFKM (55028‐1‐AP; Proteintech), anti‐E2F4 (10923‐1‐AP; Proteintech) and anti‐β‐actin (20536‐1‐AP; Proteintech).

### 2.7 Immunofluorescence

Adherent cultures on NEST coverslips (NEST Biotechnology Co.) underwent fixation for 20 min in 4% paraformaldehyde. After permeabilization with  .25% Triton X‐100, 5% bovine serum albumin was applied for a 1‐h blocking step. The primary antibodies listed for immunoblotting were incubated overnight at 4°C. Fluorescent secondary antibodies were then applied for 1 h at room temperature. A 5‐min DAPI stain preceded image acquisition on a PerkinElmer confocal laser‐scanning microscope (USA).

### 2.8 Plasmids, small interfering RNAs, lentiviral construction and cell transfection

Tsingke Biotechnology Co. prepared the HA‐AARS1, Flag‐PHGDH and Flag‐PHGDH‐K394R constructs. Delivery of the indicated plasmids used Lipofectamine 3000 (Invitrogen, Thermo Fisher Scientific) under the supplied protocol. Subsequent assays used cells harvested 48‒72 h after transfection.

The small interfering RNAs (siRNAs) were supplied by Tsingke Biotechnology Co. Huh7 and SK‐hep1 cultures received these reagents using Lipofectamine 3000 (Invitrogen, Thermo Fisher Scientific), with transfection conditions following the product instructions. The sequences were—siAARS1‐1: 5′‐GCAGTGAGATCCACTACGA‐3′; siAARS1‐2: 5′‐GTTTGGCATTCCCATTGAA‐3′; siE2F4‐1: 5′‐GCAAGAACUAGACCAGCACAATT‐3′; siE2F4‐2: 5′‐GACCUCUUUGAUGUGCCUGUUTT‐3′.

Shanghai GeneChem Co. supplied the lentiviral preparations for AARS1 depletion and overexpression. The short hairpin RNA (shRNA) construction sequences were—shAARS1‐1: 5′‐CCGGCGATGTCCAGAAACGAGTGTTCTCGAGAACACTCGTTTCTGGACATCGTTTTTG‐3′ (forward), 5′‐AATTCAAAAACGATGTCCAGAAACGAGTGTTCTCGAGAACACTCGTTTCTGGACATCG‐3′ (reverse); shAARS1‐2: 5′‐CCGGGCAGAATAAGATGTCCAACTACTCGAGTAGTTGGACATCTTATTCTGCTTTTTG‐3′ (forward), 5′‐AATTCAAAAAGCAGAATAAGATGTCCAACTACTCGAGTAGTTGGACATCTTATTCTGC‐3′ (reverse); shPHGDH‐1: 5′‐CCGGCTTCGATGAAGGACGGCAAATCTCGAGATTTGCCGTCCTTCATCGAAGTTTTTG‐3′ (forward); 5′‐AATTCAAAAACTTCGATGAAGGACGGCAAATCTCGAGATTTGCCGTCCTTCATCGAAG‐3′ (reverse); shPHGDH‐2: 5′‐CCGGCAGCAATAACCGTCTAATAAACTCGAGTTTATTAGACGGTTATTGCTGTTTTTG‐3′ (forward); 5′‐AATTCAAAAACAGCAATAACCGTCTAATAAACTCGAGTTTATTAGACGGTTATTGCTG‐3′ (reverse). Stable Huh7 and SK‐hep1 populations were generated by 48 h of exposure to the appropriate lentiviral particles, followed by puromycin selection at 2 µg/mL. AARS1 depletion or overexpression was checked by quantitative reverse‐transcription polymerase chain reaction (qRT‐PCR) or Western blotting.

### 2.9 Cell Counting Kit‐8 assay

For Cell Counting Kit‐8 (CCK‐8) measurements, each well of a 96‐well plate received 1000 cells. The assay reagent (40203ES80; Yeasen Biotechnology) was used under the supplied protocol at 0, 24, 48, 72 and 96 h. A multimode plate reader recorded the 450‐nm absorbance as the cell‐viability readout.

### 2.10 Colony formation assay

Clonogenic cultures started wih 1000 cells in each well of a six‐well plate. Colonies were processed once they were visible without magnification and contained at least 50 cells. Processing consisted of fixation in 4% paraformaldehyde for 15 min and subsequent crystal violet staining.

### 2.11 Wound healing assay

Monolayers in six‐well plates were scratched at 80%‒90% confluence using a sterile pipette tip. After creating the straight wound, cultures were maintained in DMEM with 1% FBS. The wound was photographed immediately and again at 24 and 48 h.

### 2.12 Transwell assay

Migration and invasion were assessed in uncoated and Matrigel‐coated Transwell inserts, respectively. All other assay conditions were identical. The lower compartments contained DMEM with 20% FBS. Cell suspensions in DMEM were loaded into the upper inserts and maintained at 37°C until cells reached the membrane underside. Inserts received two saline washes, fixation in 4% paraformaldehyde, and crystal violet staining. Cells remaining on the upper face, together with residual Matrigel in the invasion assay, were removed. Cells on the underside were then counted by inverted phase‐contrast microscopy.

### 2.13 Flow cytometric analysis of cell apoptosis

Apoptosis induction used recombinant human TNF‐α at 10 ng/mL for 24 h. Harvested cells were rinsed twice in cold PBS and labeled with the Annexin V‐APC/propidium iodide (PI) kit following its instructions. Labelling proceeded for 15‒20 min at room temperature with light excluded. Flow cytometry was then used to assess the stained populations.

### 2.14 Animal studies

Animal work followed the Guidelines for the Care and Use of Laboratory Animals of China and was authorized by the Laboratory Animal Ethics Committee of Nanfang Hospital (IACUC‐LAC‐20250320‐008). The Animal Experimental Center of Southern Medical University supplied male BALB/c nude mice aged 4‒5 weeks. AARS1 xenografts were established by subcutaneous implantation of 5 × 10^6^ Huh7 cells carrying lentiviral control or AARS1‐targeting shRNA. The lactate‐sensing and lactyltransferase functions reported by Zong et al.^9^ motivated a test of whether exogenous lactate could still enhance tumour growth after AARS1 depletion. Both cell‐derived xenograft groups received either PBS or sodium L‐lactate (1 g/kg; Sigma‒Aldrich, Cat. No. 71718) by daily intraperitoneal injection. Dosing began 7 days before inoculation and continued for the duration of the study. Tumour volumes were recorded at 7‐day intervals, and animals were sacrificed 21 days after inoculation.

For the PHGDH rescue study, nude mice received subcutaneous injections of PHGDH‐depleted cells re‐expressing PHGDH‐WT or PHGDH‐K394R. Tumour dimensions were recorded at the indicated times, with volume derived from length × width^2^/2. Excised tumours were photographed and weighed at the endpoint.

The β‐alanine study compared control and treatment groups of nude mice carrying subcutaneous Huh7 xenografts, with five animals per group. Drinking water contained 1.2% (w/v) β‐alanine (A606168; Sangon Biotech) and was renewed every week. In addition, each treated mouse received 200 µL of this solution by oral gavage three times weekly. Growth was followed during treatment, and excised tumours were weighed when the experiment ended.

### 2.15 Immunoprecipitation and co‐immunoprecipitation assays

Association between AARS1 and PHGDH was examined by exogenous co‐immunoprecipitation (co‐IP) after transfection with HA‐AARS1 and/or Flag‐PHGDH. Separate transfections with Flag‐PHGDH or Flag‐PHGDH‐K394R were used for immunoprecipitation (IP)‐based lactylation analysis. Sodium lactate (NaLa) was applied before harvest where indicated. Transfected cells were extracted in IP buffer containing protease inhibitors. Equal protein inputs were incubated with anti‐HA or anti‐Flag magnetic beads using the supplier's procedure. After thorough washing, bead‐bound material was released by boiling in SDS loading buffer and analyzed by immunoblotting for the indicated targets.

### 2.16 PHGDH activity assay

Whole‐cell extracts provided the material for the PHGDH colorimetric activity assay (ab273328). Plate‐reader absorbance values were converted to enzyme activity using the kit procedure. Each value was normalized against the total protein concentration of its corresponding lysate; immunoprecipitated protein was not used for this activity measurement.

### 2.17 GSH quantification

Cellular GSH was assayed with the Beyotime GSH and GSSG kit (S0053), following the supplied procedure. The assay signal was read at 412 nm on a SpectraMax 190 plate reader (Molecular Devices).

### 2.18 Intracellular reactive oxygen species detection

Reactive oxygen species (ROS)‐associated fluorescence was measured with the 2',7'‐dichlorodihydrofluorescein diacetate (DCFH‐DA) kit S0033 (Beyotime). Following treatment, Huh7 and SK‐hep1 cultures were loaded with DCFH‐DA for 20 min at 37°C without light exposure. PBS washing preceded fluorescence microscopy. 2',7'‐dichlorofluorescein (DCF) intensity provided the relative intracellular ROS readout, following the kit protocol.

### 2.19 Lactate measurement

Lactate was quantified with kit A019‐2‐1 (Nanjing Jiancheng Bioengineering Institute). The assay was run and calculated according to the kit protocol, using plate‐reader absorbance at 530 nm. Cell experiments used culture supernatants collected after treatment, with values normalized to the corresponding cell count. For clinical HCC specimens, tissue homogenates were assayed and normalized by sample protein concentration.

### 2.20 Fluorescence‐based extracellular acidification assay

Treated Huh7 and SK‐hep1 cells were analyzed with the fluorescence‐based ECAR kit E‐BC‐F069 (Elabscience). After incubation with the supplied reagents, fluorescence was recorded on a plate reader using 490‐nm excitation and 535‐nm emission. Acidification was evaluated under the kit protocol and expressed relative to the corresponding cell number.

### 2.21 Mass spectrometry‐based lactylome analysis

PTM Biolabs performed lactylome profiling on Huh7 cultures transfected with siNC or siAARS1. Extracted proteins were digested, and the resulting peptide mixtures underwent affinity enrichment with antibodies recognizing lactyl‐lysine. Enriched material was analyzed by liquid chromatography‒tandem mass spectrometry (LC‒MS/MS). Processing of the spectra identified modified peptides and their lactylation sites, allowing comparison of Kla between control and AARS1‐depleted cultures.

### 2.22 Structural modelling and molecular docking

Starting structures were the AlphaFold predictions accessed through UniProt (https://www.uniprot.org/) for PHGDH (O43175) and AARS1 (P49588). Modelling of lactylated PHGDH‐K394 used PyMOL 3.1.3, Gaussian 16 and GaussView 6.0; Sobtop 1.0(dev5) generated the modified‐residue topology parameters. GROMACS 2024.3 was used for molecular dynamics of the modified protein. Preparation involved a cubic solvent box, charge neutralization with counterions and energy minimization. constant particle number, volume, and temperature (NVT) and constant particle number, pressure, and temperature (NPT) equilibration at 300 K preceded a 10‐ns production run. Superimposition and root‐mean‐square deviation (RMSD) calculations were used to compare unmodified PHGDH with the K394‐lactylated model.

ClusPro 2.0 (https://cluspro.bu.edu/) was used with its default global settings to dock AARS1 against either PHGDH model. The resulting complexes were simulated with the same molecular dynamics procedure. Trajectory‐based binding‐energy estimates used gmx_MMPBSA 1.6.4 and MM/GBSA, specifying the generalized Born model igb = 5 and  .150 M salt.

### 2.23 Targeted amino acid metabolomics analysis

Targeted metabolomic measurements compared Huh7 cells expressing PHGDH‐WT with those expressing PHGDH‐K394R, using three biological replicates per group. MetWare Biotechnology Co. extracted cellular metabolites and analyzed them by targeted LC‒MS/MS. Amino acids and related derivatives were identified and quantified through standard procedures. Normalized relative abundances provided the basis for differential analysis between the two PHGDH groups.

### 2.24 Statistical analyses

Analyses used IBM SPSS 20.0, GraphPad Prism 5 or R, as specified for individual datasets. Unless indicated otherwise, quantitative summaries report means with standard deviation, and cell experiments comprise three independent biological replicates. A biological replicate denotes a separately conducted experiment, not multiple readings from one sample. Figure legends identify the relevant numbers of experiments, patients or animals. For two groups, matched observations were evaluated by paired Student's *t*‐tests and independent observations by unpaired Student's *t*‐tests. Comparisons spanning multiple groups or factors used one‐ or two‐way analysis of variance (ANOVA), as indicated. Public‐cohort tumour‒normal expression comparisons used the Wilcoxon rank‐sum procedure described above. Associations between continuous variables were evaluated with Pearson or Spearman correlation, as specified. Kaplan‒Meier estimates and log‐rank comparisons were used for overall survival. Univariate and multivariate Cox proportional hazards models yielded hazard ratios (HRs) with 95% confidence intervals. Testing was two‐sided throughout, with *p* < .05 defining statistical significance.

## 3 RESULTS

### 3.1 AARS1 is upregulated in HCC, is associated with poor prognosis, and is positively regulated by E2F4

The three public cohorts showed a consistent relationship between AARS1 and HCC. In The Cancer Genome Atlas (TCGA), GEO‐GSE14520 and ICGC, tumour tissue contained significantly more AARS1 mRNA than non‐tumour tissue (Figure 1A). Higher expression also identified groups with shorter overall survival in Kaplan‒Meier comparisons: *p* < .001 for TCGA, *p* = .045 for GSE14520, and *p* = .010 for ICGC (Figure 1B).

**FIGURE 1:**
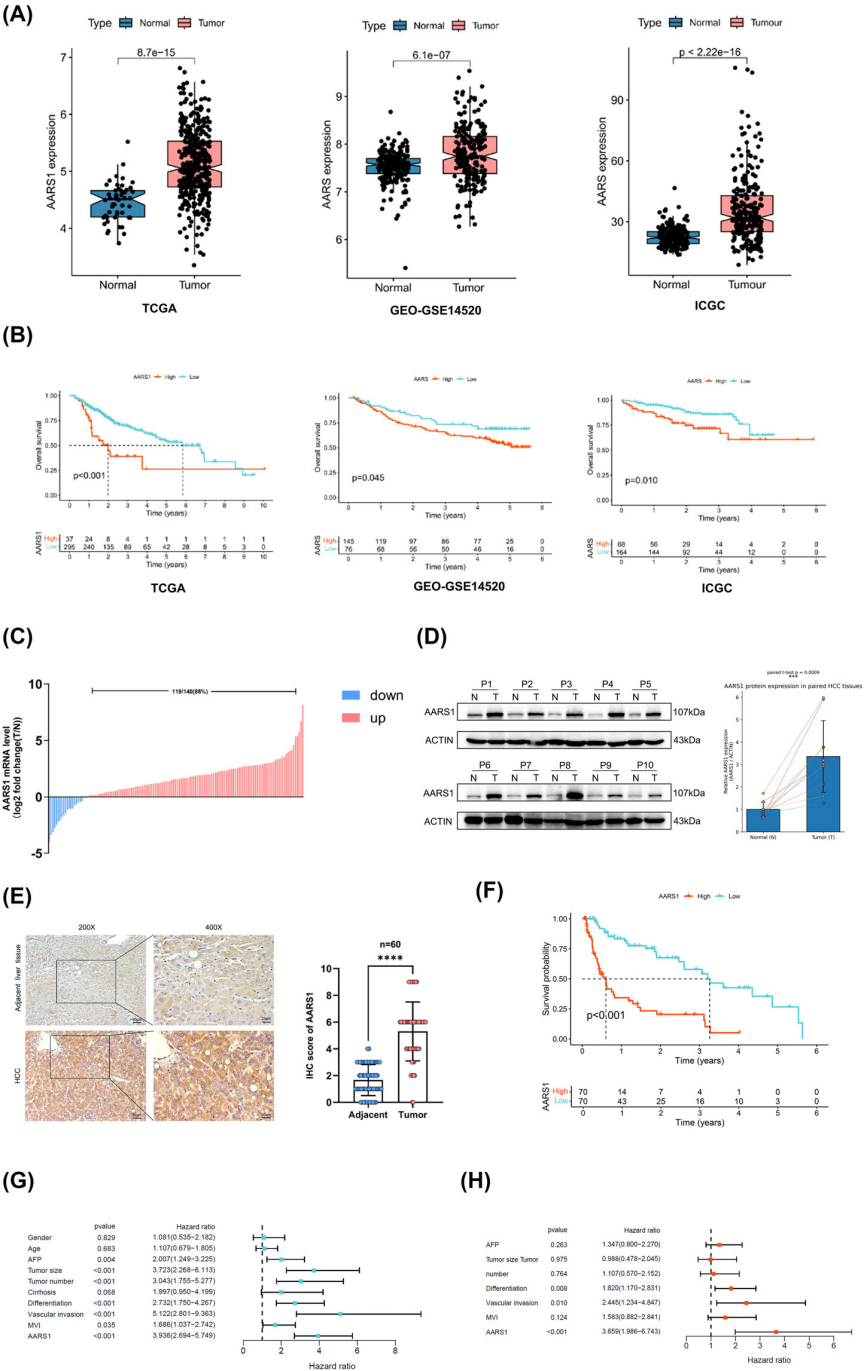
Alanyl‐tRNA synthetase 1 (AARS1) is highly expressed in hepatocellular carcinoma (HCC) and associated with poor prognosis. (A) Tumour‒normal comparisons of AARS1 expression in The Cancer Genome Atlas (TCGA), Gene Expression Omnibus (GEO) and International Cancer Genome Consortium (ICGC). (B) Survival curves for the high‐ and low‐expression groups within those public cohorts. (C) AARS1 transcript measurements in clinical tumour (T) and adjacent normal (N) specimens. (D) Immunoblot assessment of AARS1 protein in the corresponding tissue categories. (E) Example AARS1 immunohistochemistry (IHC) images and staining scores from the institutional HCC tissue collection. (F) Kaplan‒Meier survival estimates stratified by AARS1 in the institutional cohort. (G and H) Cox analyses evaluating AARS1 and other clinical variables, without adjustment in (G) and with multivariable adjustment in (H). Public‐cohort expression comparisons in (A) used the Wilcoxon rank‐sum test; survival comparisons in (B) and (F) used the log‐rank test. Panels (G) and (H) show univariate and multivariate Cox regression, respectively. Tissue‐comparison procedures are described in Statistical analyses. Significance notation: ^*^ *p* < .05, ^**^ *p* < .01, ^***^ *p* < .001 and ^****^ *p* < .0001.

Clinical tissue measurements supported the public‐data findings. AARS1 mRNA was higher in the tumour member of 119 of 140 tissue pairs (85%; Figure 1C). Immunoblot analysis of 10 pairs showed a corresponding protein increase (*p* = .0009; Figure 1D). IHC likewise showed stronger tumour staining and higher scores than in adjacent non‐tumour tissue (Figure 1E). Thus, the clinical material provided both RNA‐ and protein‐level evidence of AARS1 upregulation.

In our clinical cohort, the high‐AARS1 group had shorter survival (*p* < .001; Figure 1F). The univariate Cox estimate for high expression was HR = 3.936 (95% confidence interval [CI]: 2.694‒5.749; *p* < .001; Figure 1G). Adjustment for the other clinicopathological variables retained this adverse association, with HR = 3.659 (95% CI: 1.986‒6.743; *p* < .001; Figure 1H). These observations support further evaluation of AARS1 as a prognostic marker.

Intersecting the hTFtarget, ChEA, GTRD and ChIP‐Atlas predictions yielded 12 candidate transcription factors upstream of AARS1 (Figure S1A). E2F4 had the strongest positive correlation with AARS1 among these candidates in TCGA‐LIHC (Spearman's *ρ* = .59; Figure S1B). The corresponding scatterplot showed *R* = .60 and *p* = 7.6 × 10^‒38^ (Figure S1C). Two independent E2F4 siRNAs lowered both AARS1 transcript and protein in Huh7 and SK‐hep1 cells (Figure S1D,E). Together, the association and perturbation results identify E2F4 as a candidate positive regulator of AARS1.

### 3.2 AARS1 promotes HCC progression in vitro and in vivo

Endogenous expression guided the selection of models for functional studies. Across the cell‐line panel, immunoblotting showed comparatively abundant AARS1 in Huh7 and SK‐hep1 and lower levels in Hep3B and SNU‐449 (Figure S2A). We therefore used two independent shRNAs for depletion in the former pair and introduced AARS1 overexpression in the latter. Transcript and protein measurements verified the respective interventions (Figures 2A,B and S2B,C).

**FIGURE 2:**
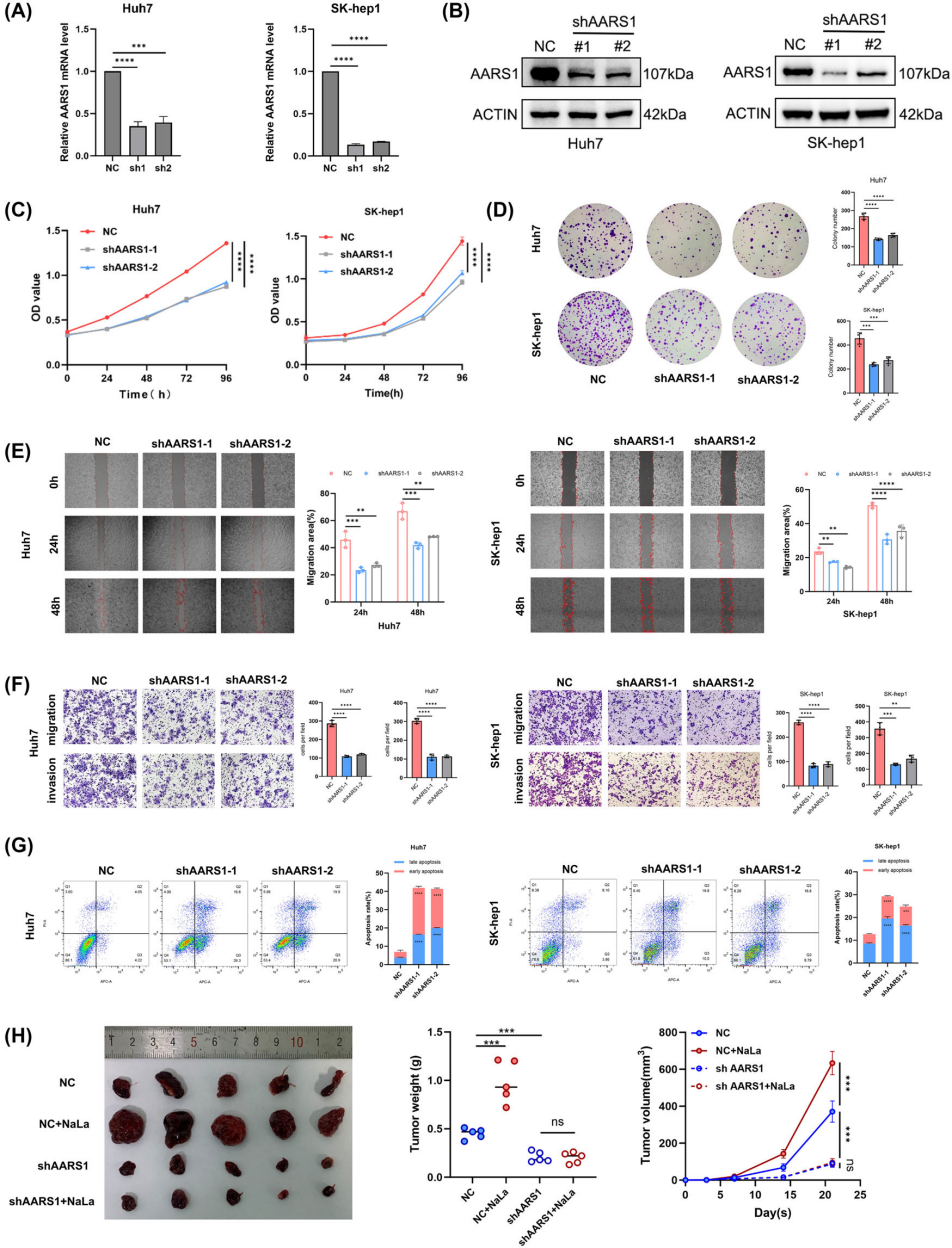
Alanyl‐tRNA synthetase 1 (AARS1) promotes hepatocellular carcinoma (HCC) progression in vitro and in vivo. (A and B) AARS1 transcript measurements (A) and example protein blots (B) after control short hairpin RNA (shRNA) (negative control, NC), shAARS1‐1 or shAARS1‐2 transduction in Huh7 and SK‐hep1. ACTIN provides the loading reference. (C) Cell Counting Kit‐8 (CCK‐8) growth measurements over the indicated interval. (D) Colony images with colony‐count analysis. (E) Wounds photographed at 0, 24 and 48 h, with closure quantified at the latter two times. (F) Transwell migration and invasion images with counts per field. (G) Annexin V‐APC/propidium iodide (PI) flow‐cytometry plots and the measured early‐ and late‐apoptotic fractions. (H) Excised tumour images, growth trajectories and endpoint masses for Huh7 xenografts carrying NC or shAARS1‐1. Phosphate‐buffered saline (PBS) or sodium lactate (NaLa) (1 g/kg) was given daily during the 7‐day pretreatment period before implantation. Volumes were recorded at the plotted time points and tumour masses at termination; each group contained five mice. Cell‐based findings represent three independently conducted experiments. Numerical summaries give means and standard deviation (SD); comparisons used two‐tailed Student's *t*‐tests or two‐way analysis of variance (ANOVA) as appropriate. Symbols denote ^*^ *p* < .05, ^**^ *p* < .01, ^***^ *p* < .001 and ^****^ *p* < .0001; ns indicates *p* > .05. OD, optical density.

The functional assays showed opposite responses to depletion and overexpression. Removing AARS1 inhibited Huh7 and SK‐hep1 growth in CCK‐8 assays, while increasing AARS1 enhanced Hep3B and SNU‐449 growth (Figures 2C and S2D). Colony counts followed the same pattern (Figures 2D and S2E). Wound closure at 24 and 48 h was significantly reduced after knockdown and accelerated by overexpression (Figures 2E and S2F). Transwell counts likewise decreased for both migration and invasion after depletion and increased with overexpression (Figures 2F and S2G). Apoptosis changed in the reverse direction: it increased after knockdown and decreased after overexpression (Figures 2G and S2H). These findings link AARS1 to growth, colony formation, motility, invasion and resistance to apoptosis in the tested models.

We next asked whether the growth response to supplemental lactate requires AARS1. This question followed reports of AARS1 acting as a lactate sensor and lactyltransferase.^9^ Control and AARS1‐depleted Huh7 xenografts were compared under PBS or NaLa treatment. Depletion reduced growth and final tumour weight. NaLa increased volume and weight in control tumours, but neither measure rose significantly in the depleted group (Figure 2H). Supplemental lactate therefore did not restore the growth lost after AARS1 knockdown. This pattern is consistent with AARS1 contributing to lactate‐associated tumour growth and linking lactate availability with protein lactylation.

### 3.3 AARS1 regulates global protein lactylation and promotes lactate production in HCC

Reports associating HCC lactylation with unfavourable prognosis motivated examination of our clinical material.^8^ Tumour samples showed greater global Kla than paired adjacent non‐tumour samples by both immunoblotting and IHC (Figure 3A,B). Cellular imaging localized Kla signals to the cytoplasm as well as the nucleus (Figure S3A).

**FIGURE 3:**
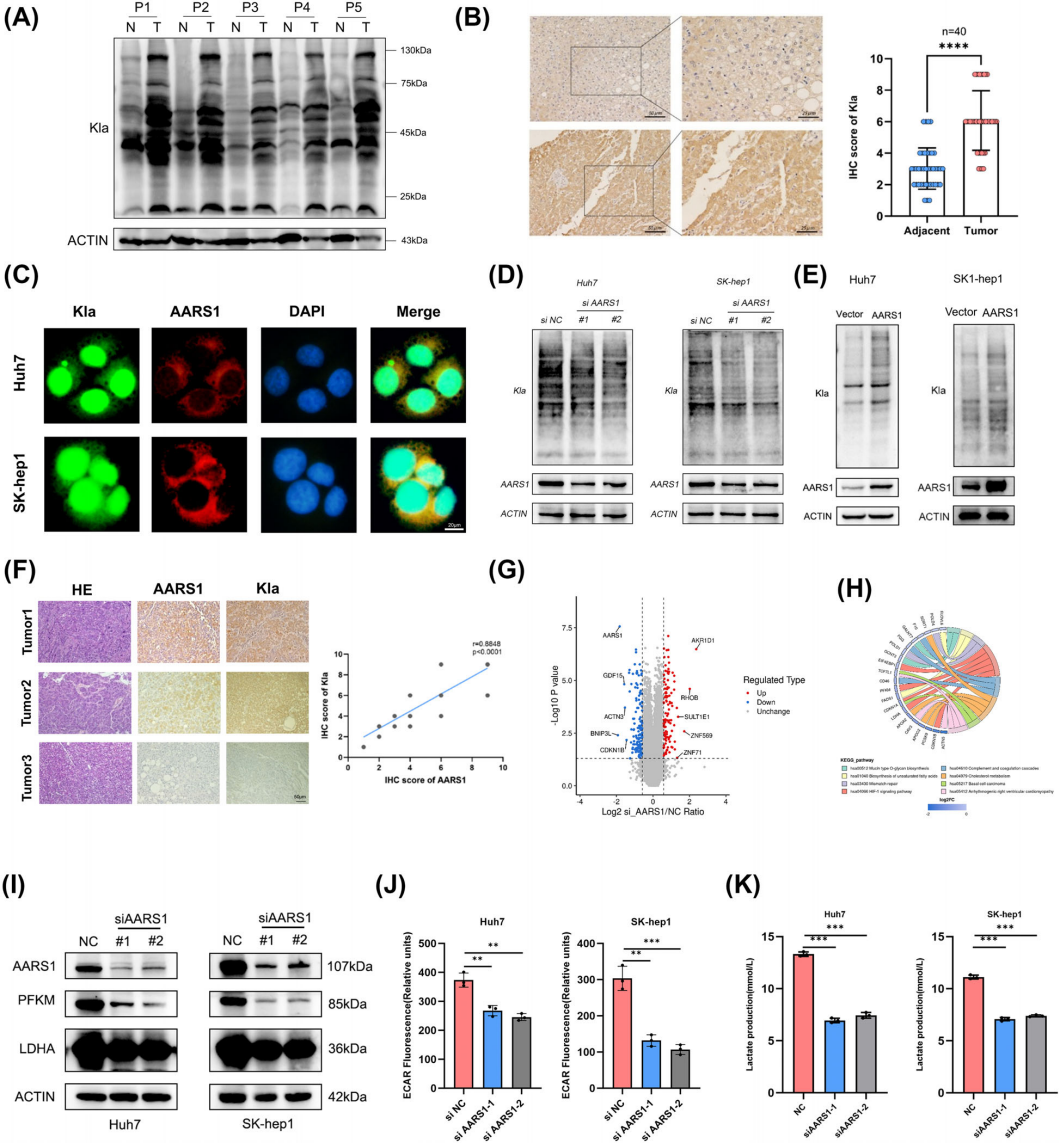
Alanyl‐tRNA synthetase 1 (AARS1) regulates global protein lactylation and promotes lactate production in hepatocellular carcinoma (HCC). (A) Immunoblot detection of lysine lactylation (Kla) in paired tumour (T) and adjacent non‐tumour (N) specimens. (B) Tissue Kla staining examples and immunohistochemistry (IHC) scores for paired HCC and adjacent normal tissues (*n* = 40; scale bar: 50 µm). (C) AARS1 and Kla fluorescence in Huh7 and SK‐hep1 (scale bar: 20 µm). (D and E) Global Kla measurements in these two cell lines following AARS1 depletion (D) or overexpression (E). (F) Haematoxylin and eosin (H&E) and AARS1/Kla immunohistochemistry (IHC) images from HCC specimens, together with the relationship between AARS1 expression and Kla staining. (G) Proteomic volcano plot comparing siAARS1‐treated Huh7 with scrambled small interfering RNA (siRNA) controls (NC). (H) Connections between altered proteins and enriched KEGG pathways, shown as a chord diagram; link colour encodes protein log_2_FC. (I) phosphofructokinase, muscle type (PFKM) and lactate dehydrogenase A (LDHA) immunoblots after AARS1 depletion in Huh7 and SK‐hep1. (J) Acidification‐associated fluorescence in the same models after depletion, reported in relative fluorescence units. (K) Lactate measurements following AARS1 knockdown in both cell lines. Numerical summaries report means with standard deviation (SD). Panel (B) uses a paired two‐tailed Student's *t*‐test, whereas (J) and (K) use the unpaired two‐tailed form. Pearson correlation evaluates the association in (F). Symbols correspond to ^*^ *p* < .05, ^**^ *p* < .01, ^***^ *p* < .001 and ^****^ *p* < .0001.

The reported cytoplasmic lactyltransferase function of AARS1^9^, ^11^ led us to examine its relationship with global Kla. AARS1 and Kla fluorescence overlapped in the cytoplasm (Figure 3C). In both Huh7 and SK‐hep1, reducing AARS1 lowered global Kla (Figure 3D), whereas overexpressing it raised Kla (Figure 3E). Clinical IHC scores also linked AARS1 expression positively with Kla staining (*r* = .8848, *p* < .0001; Figure 3F). These observations support AARS1‐associated regulation of protein lactylation in HCC.

Because AARS1 uses lactate and ATP during lactylation,^9^ we also examined whether AARS1 perturbation changes lactate production. Proteomics after AARS1 depletion in Huh7 identified decreases in multiple proteins, including PFKM and LDHA; Kyoto Encyclopedia of Genes and Genomes (KEGG) mapping placed these enzymes in the HIF‐1 signalling pathway (Figure 3G,H). Their roles in glycolysis and lactate generation^23^ prompted immunoblot assessment, which showed lower PFKM and LDHA signals after depletion in Huh7 and SK‐hep1 (Figure 3I). Both cell lines also showed significantly lower fluorescence‐based extracellular acidification (Figure 3J), and lactate measurements decreased in AARS1‐depleted cultures (Figure 3K). In 40 clinical HCC specimens, AARS1 expression correlated positively with lactate levels (Figure S3B).

The cellular measurements connect AARS1 with global Kla, lactate generation and glycolysis‐related activity. The accompanying PFKM and LDHA changes suggest possible contributions from these enzymes to the metabolic effects of AARS1 depletion.

### 3.4 Global landscape of the AARS1‐regulated lactylome in HCC cells

LC‒MS/MS profiling compared control Huh7 cells with AARS1‐depleted cells to map the associated lactylome (Figure 4A). The analysis detected 9677 proteins, including 2743 with Kla (Figure 4B). A single Kla site was found in 1189 of the modified proteins, representing 43% (Figure 4C). Modified proteins tended to have higher abundance than the proteome overall, a feature that may also affect peptide detectability (Figure 4D). Residues flanking the modified lysines were enriched for basic R and K and depleted for acidic D and E (Figure 4E).

**FIGURE 4:**
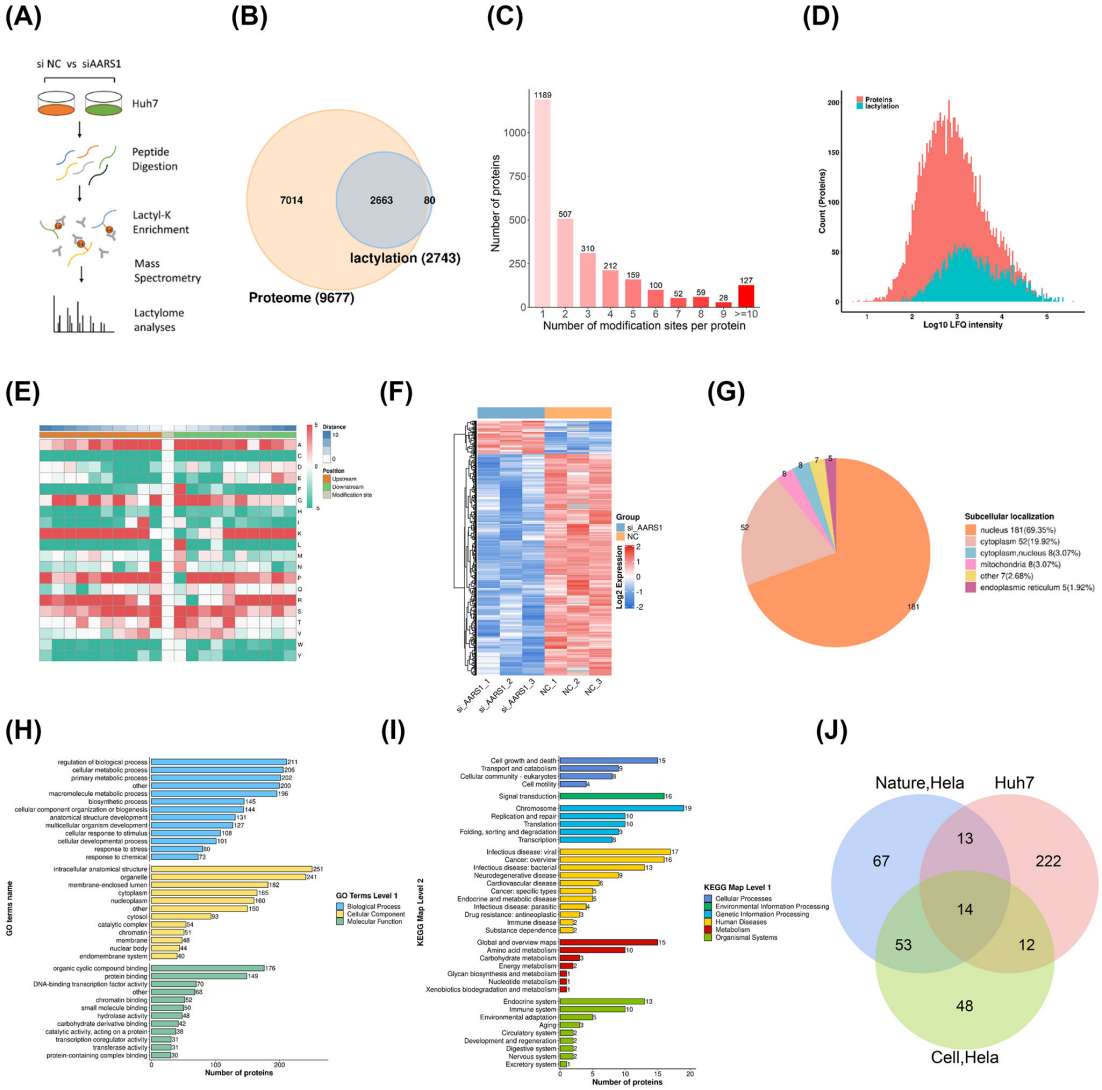
Global landscape of the alanyl‐tRNA synthetase 1 (AARS1)‐regulated lactylome in hepatocellular carcinoma (HCC) cells. (A) Workflow for antibody enrichment of lactylated peptides and subsequent mass spectrometry. (B) Counts of all detected proteins and the lactylated subset. (C) Frequency distribution of AARS1‐responsive lysine lactylation (Kla) sites per protein. (D) Label‐free quantification (LFQ) intensity distributions for protein abundance and the lactylated subset. (E) Sequence motifs associated with AARS1‐responsive lactylation. (F) Heatmap comparing the Huh7 lactylome after siAARS1 or control small interfering RNA (siRNA) transfection. (G) Donut plots of cellular compartments assigned to the affected lactylated proteins. (H and I) Functional enrichment of these proteins using Gene Ontology (GO) (H) and KEGG (I). (J) Shared and system‐specific AARS1‐responsive Kla proteins across the compared cell systems.

Relative to controls, AARS1 depletion lowered 374 Kla sites on 261 proteins and increased 56 sites on 50 proteins (Figures 4F and S4A). The affected lactylated proteins were assigned mainly to the nucleus (69%) and cytoplasm (19%; Figure 4G). Gene Ontology (GO) and KEGG enrichment linked them to amino acid, carbohydrate and energy metabolism, among other metabolic processes (Figure 4H,I).

Comparison with two published HeLa datasets^9^, ^10^ separated potentially conserved substrates from cell‐context‐associated targets. The Venn analysis contained 222 proteins unique to Huh7, 168 unique to HeLa, and 39 shared between the systems (Figure 4J). This distribution suggests a common subset of AARS1‐responsive lactylated proteins alongside a substantial cell‐type‐specific component.

### 3.5 AARS1 mediates PHGDH lactylation at K394 in HCC

Amino acid metabolism emerged from the enrichment analyses of AARS1‐responsive lactylation (Figure 4H,I). PHGDH, the serine‐synthesis enzyme, showed a significant decrease in K394 lactylation after AARS1 knockdown (Figure 5A). LC‒MS/MS assigned the modification to K394, which maps to the annotated PGDH_inter domain (Figure 5B,C). A STRING interaction network also nominated PHGDH as a potential downstream effector (Figure S5A).^24^ We then used anti‐AARS1 IP‒MS in Huh7 to identify candidate‐associated proteins. PHGDH occurred both in this dataset and among the potentially AARS1‐regulated lactylated proteins (Figure S5B). Its identification was supported by the peptide spectrum AGTGVDNVDLEAATR (Figure S5C). These converging observations motivated experiments on AARS1‒PHGDH association and PHGDH lactylation.

**FIGURE 5:**
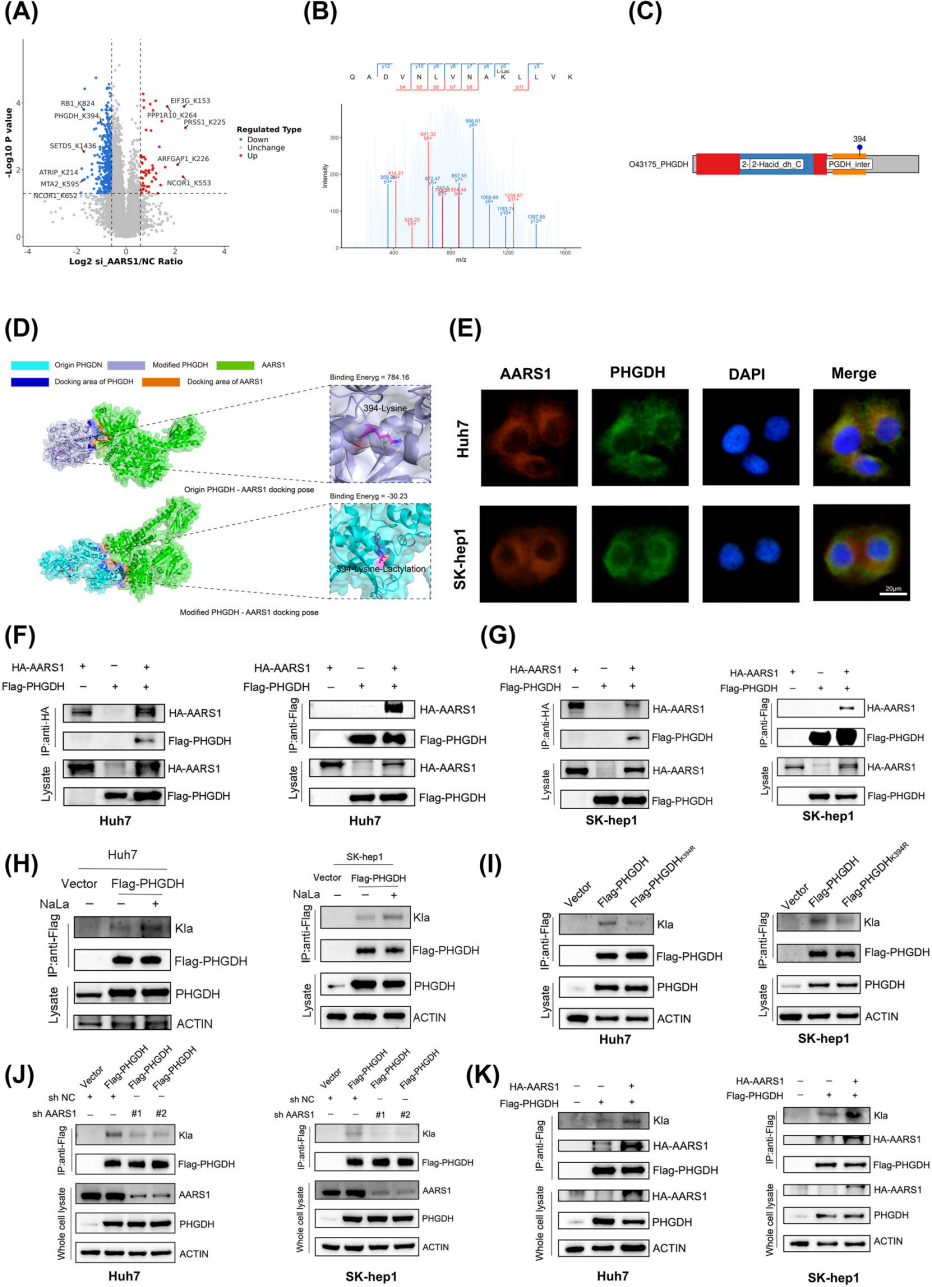
Alanyl‐tRNA synthetase 1 (AARS1) mediates lactylation of phosphoglycerate dehydrogenase (PHGDH) at K394 in hepatocellular carcinoma (HCC). (A) Volcano representation of lactylome differences between control‐small interfering RNA (siRNA) and si‐AARS1 Huh7 cells. (B) PHGDH peptide MS/MS spectra used to identify and quantify K394 lactylation. The b series retains N‐terminal peptide fragments, whereas the y series retains C‐terminal fragments. (C) Domain map locating K394 in PHGDH. (D) Computational docking of AARS1 with PHGDH. (E) Cellular fluorescence images assessing AARS1‒PHGDH colocalization. (F and G) Recovery of exogenous AARS1 and PHGDH by co‐immunoprecipitation (co‐IP) in Huh7 and SK‐hep1. (H) PHGDH lysine lactylation (Kla) signals in stable Flag‐PHGDH cultures of these cell lines, with or without sodium lactate (NaLa). (I) Comparison of Kla signals from stable Flag‐PHGDH and Flag‐PHGDH‐K394R cultures. For (H) and (I), anti‐Flag magnetic‐bead immunoprecipitation (IP) from whole‐cell extracts preceded Kla immunoblotting. (J) PHGDH Kla analysis in control‐vector or Flag‐PHGDH‐overexpressing HCC cells carrying control short hairpin RNA (shRNA) (sh NC) or AARS1 shRNAs. (K) PHGDH Kla analysis after transfection with the indicated plasmids. For (J) and (K), whole‐cell extracts underwent anti‐Flag magnetic‐bead IP and immunoblotting for the displayed targets.

Computational docking suggested candidate AARS1‒PHGDH interfaces and a possible influence of K394 lactylation on their association (Figure 5D). In Huh7 and SK‐hep1, fluorescence imaging showed cytoplasmic colocalization (Figure 5E). Reciprocal co‐IP recovered the proteins together in each cell line, providing additional evidence of cellular association (Figure 5F,G).

Anti‐Kla immunoblotting of immunoprecipitated PHGDH detected a lactylation signal that increased after NaLa exposure (Figure 5H). To investigate K394, we replaced lysine with arginine. This substitution preserves positive charge but prevents lysine acylation and is commonly used for such analyses.^25^, ^26^, ^27^ In both cell lines, K394R showed markedly less lactylation than wild‐type (WT) PHGDH, indicating that K394 contributes substantially to the signal under these conditions (Figure 5I). The overall acetylation signal measured on immunoprecipitated PHGDH was similar between WT and K394R (Figure S5E); thus, the lactylation decrease was not accompanied by an apparent change in total PHGDH acetylation.

PHGDH lactylation responded to AARS1 abundance in Huh7 and SK‐hep1: knockdown lowered the signal and overexpression increased it (Figure 5J,K). We also compared WT AARS1 with the 5A variant containing M46A/R77A/N216A/D239A/G241A substitutions in the lactyltransferase‐associated region. In depleted cells co‐expressing Flag‐PHGDH, reconstitution with 5A supported less PHGDH lactylation than reconstitution with WT AARS1 (Figure S5D). This result implicates that region in the cellular regulation of PHGDH lactylation. The combined experiments support dependence on AARS1 and identify K394 as a major PHGDH lactylation site in these HCC models.

### 3.6 AARS1‐mediated PHGDH‐K394 lactylation enhances enzymatic activity, supports serine metabolism, and alleviates oxidative stress

Prior HCC profiling described increased serine metabolism despite reduced expression of PHGDH,^17^ suggesting that abundance is an incomplete measure of its metabolic contribution. Known PHGDH modifications—including phosphorylation, ubiquitination, acetylation and methylation—can alter function and influence tumour progression.^22^ The contribution of lactylation is less clear. We first examined clinical relevance in five tumour/non‐tumour tissue pairs. PHGDH IP followed by immunoblotting showed stronger tumour‐associated lactylation signals together with higher AARS1 levels (Figure 6A). We then investigated how this modification relates to enzyme activity and downstream metabolism.

**FIGURE 6:**
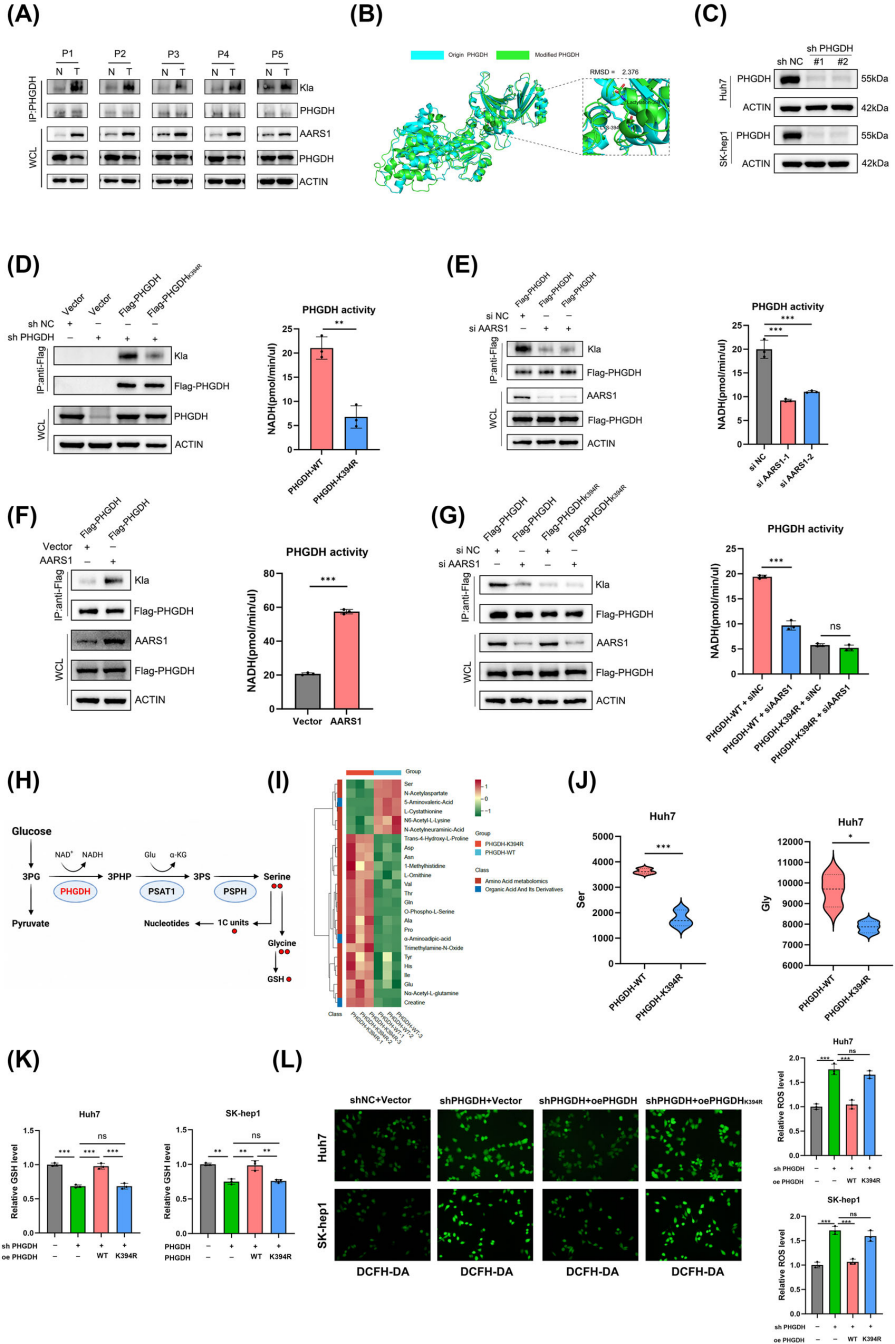
K394 lactylation of phosphoglycerate dehydrogenase (PHGDH) enhances its enzymatic activity, promotes serine biosynthesis and alleviates oxidative stress. (A) PHGDH‐associated lysine lactylation (Kla) in five paired clinical tumours (T) and adjacent non‐tumour tissues (N), with P1‒P5 identifying patients. Endogenous PHGDH was recovered by anti‐PHGDH immunoprecipitation (IP) and probed for Kla and PHGDH; whole‐cell lysates (WCL) were probed for alanyl‐tRNA synthetase 1 (AARS1), PHGDH and ACTIN. (B) Superimposed unmodified (cyan) and K394‐lactylated (green) PHGDH models, with root‐mean‐square deviation (RMSD) = 2.376; the enlargement shows the region around K394. (C) Verification of PHGDH depletion by two independent short hairpin RNAs (shRNAs) in Huh7 and SK‐hep1, using ACTIN as the loading reference. (D) PHGDH‐depleted Huh7 reconstituted with Flag‐PHGDH‐WT or Flag‐PHGDH‐K394R. Anti‐Flag magnetic‐bead IP was followed by Kla and Flag immunoblotting. (E and F) Flag‐PHGDH‐reconstituted, PHGDH‐depleted Huh7 receiving control or either of two AARS1 small interfering RNAs (siRNAs) (E), or empty vector versus an AARS1 construct (F). IP‐derived Flag‐PHGDH was probed for Kla and Flag. (G) Wild‐type (WT)‐ or K394R‐reconstituted, PHGDH‐depleted Huh7 receiving control or AARS1 siRNA, with IP material probed for Kla and Flag. In (D)‒(G), a separate colorimetric assay measured PHGDH activity in whole‐cell lysates, normalized by the total protein concentration of each lysate. (H) Diagram connecting de novo serine synthesis with glycine, glutathione and nucleotide production. (I) Relative metabolite‐abundance heatmap from targeted profiling of WT‐ or K394R‐reconstituted, PHGDH‐depleted Huh7. (J) Intracellular Ser and Gly distributions in WT‐ and K394R‐expressing Huh7, displayed as violin plots. (K) Relative GSH in Huh7 and SK‐hep1 after PHGDH depletion and WT or K394R rescue. (L) DCFH‐DA images and relative intracellular ROS quantification for the indicated groups in both cell lines. Bars summarize means and standard deviation (SD). ^*^ *p* < .05, ^**^ *p* < .01, ^***^ *p* < .001; ns denotes no significant difference.

Superimposing the modelled unmodified and K394‐lactylated PHGDH structures gave an RMSD of 2.376 and suggested local rearrangements near K394 (Figure 6B). Functional comparisons used stable PHGDH depletion followed by re‐expression of Flag‐PHGDH‐WT or Flag‐PHGDH‐K394R, reducing the contribution of endogenous protein (Figure 6C). The mutant showed markedly lower lactylation and lower colorimetric PHGDH activity than WT (Figures 6D and S6A). Activity was measured in whole‐cell lysates and normalized against each lysate's total protein concentration. These comparisons are consistent with a contribution of K394 lactylation to catalytic activity.

Manipulating AARS1 changed both PHGDH lactylation and activity: knockdown decreased the two readouts, while overexpression increased them (Figures 6E,F and S6B,C). In the reconstitution experiment, AARS1 depletion lowered activity in the PHGDH‐WT background but produced no significant further reduction from the already low K394R activity (Figures 6G and S6D). The pattern supports a model linking AARS1‐associated K394 lactylation to PHGDH activity.

We next examined metabolites connecting PHGDH to serine synthesis and antioxidant defense. PHGDH converts 3‐phosphoglycerate into 3‐phosphohydroxypyruvate at the committed entry step of serine synthesis. Serine can then supply glycine, one‐carbon metabolism and glutathione synthesis (Figure 6H). Targeted profiling detected differences in multiple amino acid and organic acid abundances between Huh7 cells expressing WT and K394R PHGDH (Figure 6I). Serine and glycine were both significantly less abundant in the mutant group (Figure 6J), consistent with involvement of K394 lactylation in PHGDH‐associated serine metabolism.

Redox measurements showed parallel functional differences. PHGDH depletion significantly lowered reduced glutathione (GSH) in Huh7 and SK‐hep1. Reintroducing WT PHGDH restored GSH, whereas K394R did not provide effective rescue (Figure 6K). DCFH‐DA fluorescence increased after depletion, indicating greater intracellular ROS accumulation. WT re‐expression reduced this signal more effectively than K394R (Figure 6L). Together with the enzyme and metabolite measurements, these observations support a link between AARS1‐associated PHGDH‐K394 lactylation, serine/glycine metabolism, and maintenance of cellular redox balance.

### 3.7 PHGDH‐K394 lactylation promotes malignant phenotypes in HCC cells

Serine supplies one‐carbon units for nucleotide production and proliferative growth.^28^ Having connected PHGDH‐K394 with activity and serine metabolism, we tested its relationship with malignant cellular behaviour. Huh7 and SK‐hep1 with stable PHGDH knockdown were reconstituted with WT or the K394R substitution mutant. Immunoblotting verified depletion of endogenous PHGDH and similar re‐expression of the two constructs (Figure 7A).

**FIGURE 7:**
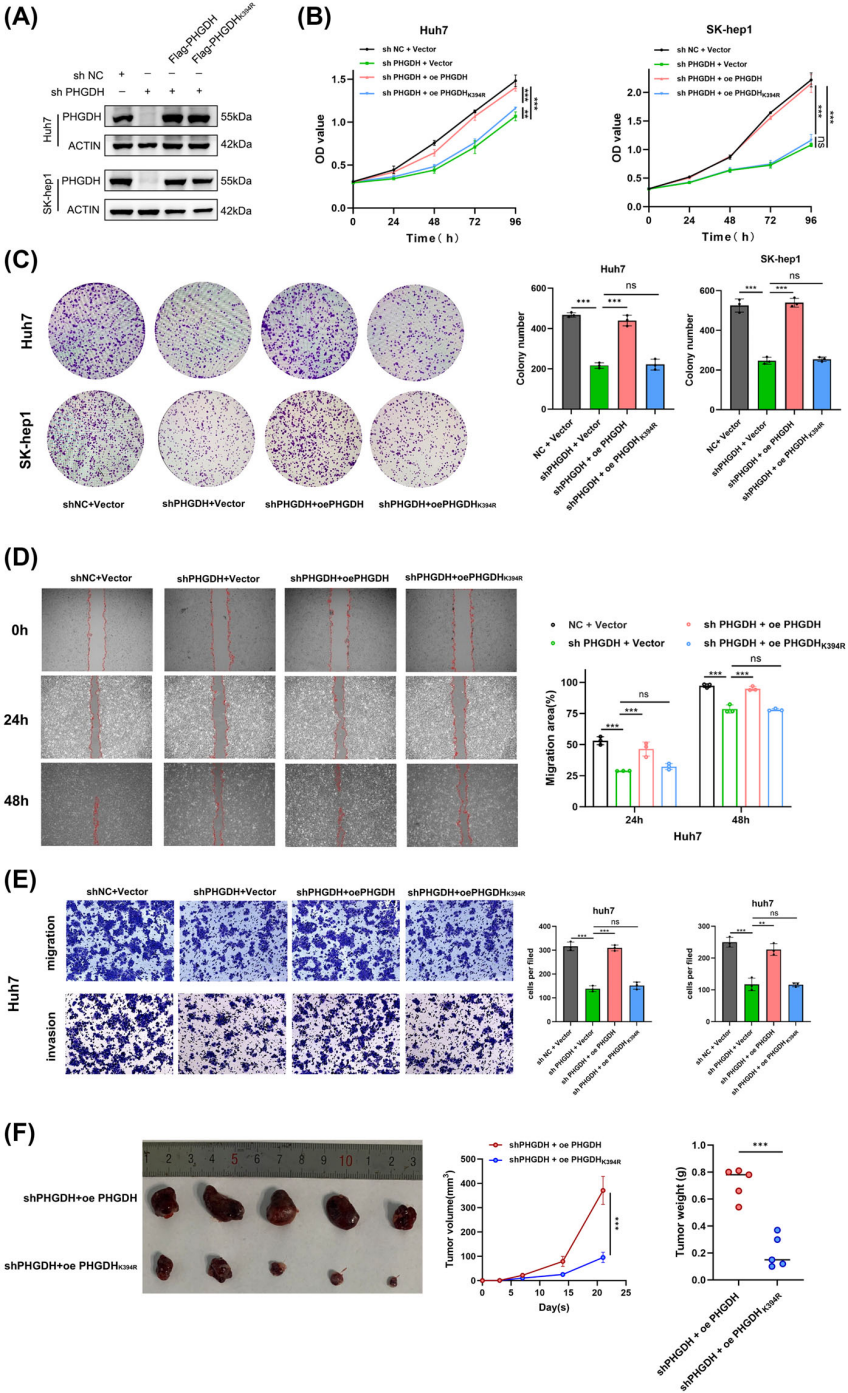
Phosphoglycerate dehydrogenase (PHGDH) K394 lactylation promotes the malignant phenotypes of hepatocellular carcinoma (HCC) cells. (A) Huh7 and SK‐hep1 received control or PHGDH short hairpin RNA (shRNA) and then shRNA‐resistant wild‐type (WT) or K394R PHGDH constructs. (B) Growth measurements for the indicated groups in both models. (C) Colonies visualized with crystal violet after 10 days. (D) Scratch‐wound migration of the indicated Huh7 groups, imaged at 0, 24 and 48 h. (E) Transwell assessment of migration and invasion in the Huh7 variants. (F) Subcutaneous xenografts established in 5‐week‐old nude mice with 3 × 10^6^ Huh7‐shPHGDH + PHGDH or Huh7‐shPHGDH + PHGDHK394R cells. Tumours were dissected and photographed after 21 days; tumour mass and volume were measured. Cell‐based findings represent three independently conducted experiments. Numerical summaries give means and standard deviation (SD). Two‐tailed Student's *t*‐tests or two‐way analysis of variance (ANOVA) were applied as appropriate. Symbols denote ^*^ *p* < .05, ^**^ *p* < .01, ^***^ *p* < .001 and ^****^ *p* < .0001; ns indicates *p* > .05.

Loss of PHGDH reduced growth and colony formation in both models. WT re‐expression largely restored these functions, while K394R provided little or no recovery (Figure 7B,C). Depletion also impaired migration and invasion in wound and Transwell assays. Here too, WT substantially reversed the defects, whereas K394R did not (Figures 7D,E and S7A,B). These rescue comparisons support a contribution of PHGDH‐K394 lactylation to proliferative and motile phenotypes.

To examine genetic dependence, we depleted AARS1 after reconstituting PHGDH‐depleted cells with WT or K394R. CCK‐8 measurements showed that AARS1 knockdown inhibited proliferation in the WT background, whereas no significant additional inhibition was detected in the mutant background (Figure S7C). Colony formation and Transwell migration showed the same pattern: decreases with WT, but no significant further decrease with K394R (Figure S7D,E). These results support participation of PHGDH‐K394 lactylation in AARS1‐associated growth and migration.

The xenograft comparison extended these findings in vivo: K394R‐expressing cells produced slower‐growing tumours with lower final weights than WT‐expressing cells (Figure 7F). We also tested β‐alanine because its reported inhibition of AARS1‐mediated lactylation^9^ offered a pharmacological approach to this pathway. Treatment of nude mice bearing Huh7 xenografts significantly reduced growth and endpoint weight relative to controls (Figure S7F). These observations provide preliminary support for investigating AARS1‐associated lactylation as a therapeutic target in HCC.

The genetic experiments link PHGDH‐K394 to malignant phenotypes and to the cellular effects of AARS1. Together with the xenograft and β‐alanine observations, they support further evaluation of this pathway as a candidate intervention target.

## 4 DISCUSSION

Our findings connect AARS1 with PHGDH lactylation, metabolic adaptation and malignant behaviour in HCC. Clinical upregulation of AARS1 accompanied poor prognosis and increased global Kla. Cellular experiments linked AARS1‒PHGDH association and K394 lactylation with lysate enzyme activity, serine/glycine abundance, GSH maintenance and reduced ROS accumulation. K394R was less effective than WT in rescuing the measured phenotypes, and AARS1 depletion did not produce a significant additional inhibitory response in the mutant background. These results are consistent with PHGDH‐K394 lactylation contributing to the tumour‐promoting functions of AARS1.

The reported increase in HCC serine metabolism without a corresponding rise in PHGDH expression^17^ points to regulation beyond abundance. Several established modifications already provide such regulation, including phosphorylation, acetylation, ubiquitination and methylation.^22^, ^29^, ^30^, ^31^, ^32^ Our results place K394 lactylation within this framework and suggest a possible explanation for the expression‒activity mismatch. Lower PFKM/LDHA signals and reduced lactate production after AARS1 depletion further raise the possibility of feedback between AARS1, glycolytic metabolism and lactylation. The causal order and directness of these events, however, remain unresolved.

Other AARS1 substrates could act alongside PHGDH. Lactylation of p53 reduces its transcriptional activity,^9^ while modification of YAP‒TEAD strengthens proliferative signalling.^11^ These processes could respectively release growth restraints and increase the biosynthetic demand met by PHGDH, although p53‐related effects would depend on TP53 status. Activation of LDHA by lactylation could increase the lactate supply available for modification of PHGDH and other proteins.^14^ Modification of STAT1 may also favour immune evasion through diminished interferon gamma (IFN‐γ) responses.^12^ AARS1 could therefore influence metabolic supply, growth signalling and immunity through several substrates. Their relative roles and cooperation within HCC have yet to be tested directly.

The vascular and immune environment offers another context for interpreting this metabolic pathway. RBM28‐associated increases in STAT3 translation and VEGFA expression support angiogenesis.^33^ Recruitment of DOT1L by RBM30 activates STAT1‒programmed death‐ligand 1(PD‐L1) transcription and favours immune evasion.^34^ AARS1‐dependent ASH2L K312 lactylation also promotes angiogenesis in HCC through VEGFA‐related transcriptional control.^35^ PHGDH‐associated metabolic adaptation might cooperate with these vascular and immune processes during tumour growth. Such cooperation cannot be assumed: increasing STAT1 abundance and modifying STAT1 by lactylation operate at distinct regulatory levels. Cellular identity, cytokine exposure and local metabolic conditions may determine their combined effects.

Several questions remain open. E2F4 depletion supports positive regulation of AARS1 but does not demonstrate direct promoter activation. ChIP‒qPCR, mutation of predicted binding sites, and AARS1 rescue could test promoter occupancy and functional dependence. Additional substrates should be investigated using quantitative lactylomics normalized to total protein abundance. Endogenous site editing, separate or combined substrate perturbations, and interaction analyses could then distinguish independent effects from cooperation.

The supplementary analysis of immunoprecipitated PHGDH found no apparent WT‒K394R difference in overall acetylation, but it did not resolve how K394 lactylation affects catalysis. Kinetic measurements and experimental structural work are needed. Likewise, [U‐^13^C_6_]glucose tracing and metabolite rescue could test whether altered serine biosynthetic flux explains the phenotypes. The nude‐mouse xenografts provide limited information about adaptive immunity. Immunocompetent orthotopic tumours and cocultures containing immune or endothelial cells would help determine how this pathway influences immune responses and angiogenesis.

The β‐alanine response in Huh7 xenografts motivates further translational investigation of AARS1‐associated lactylation. Although β‐alanine has been reported to interfere with lactate binding to AARS1,^9^ our treatment effects cannot be assigned solely to PHGDH‐K394. A useful next step would pair tumour PHGDH IP‐based lactylation measurements with activity and flux assays to relate pathway responses to growth inhibition. Exposure, safety and contributions from other substrates also remain to be established.

## 5 CONCLUSIONS

This study links AARS1‐associated PHGDH‐K394 lactylation to metabolic adaptation and malignant behaviour in HCC. Its value as a therapeutic target will depend on resolving the contributions of other AARS1 substrates and the influence of the tumour microenvironment.

## AUTHOR CONTRIBUTIONS

*Investigation, methodology, formal analysis and data curation*: Qing Zhu. *Investigation, methodology, formal analysis and data curation*: Nanyan Yang. *Methodology*: Xiangzhuo Miao, Shiting Xu, Junming Chen, Huohui Ou, Jianmin Nie and Pengyu Zhu. *Conceptualization, data curation, validation, project administration, visualization and writing—original draft*: Yinghao Fang. *Conceptualization, validation, supervision, writing—review and editing, and funding acquisition*: Dinghua Yang.

## CONFLICT OF INTEREST STATEMENT

The authors declare they have no conflicts of interest.

## ETHICS STATEMENT

This study was conducted in accordance with the Declaration of Helsinki and was approved by the Ethics Committee of Southern Medical University Southern Hospital (approval no. NFEC‐2024‐335). All experiments involving human participants, human data, or tissue samples were conducted in full accordance with relevant guidelines and regulations. Written informed consent was obtained from all patients before their involvement in the study.

## Supporting information



Supporting Information

## Data Availability

The public datasets analyzed in this study are available from The Cancer Genome Atlas (TCGA‐LIHC), the Gene Expression Omnibus (GSE14520) and the International Cancer Genome Consortium (ICGC). The omics raw data generated in this study have not yet been deposited in a public repository. Additional data supporting the findings of this study are available from the corresponding authors upon reasonable request. Access to individual‐level clinical data is subject to applicable privacy protections and institutional ethical approval.
