# Supplementary material for: AARS1‐mediated PHGDH lactylation promotes hepatocellular carcinoma progression by enhancing serine biosynthesis
Source: Clin Transl Med. 2026 Nov 14;16(11):e70899. doi: 10.1002/ctm2.70899 (PMC13770211; doi:10.1002/ctm2.70899)
Supplement: Supplementary file 1 [file CTM2-16-e70899-s001.docx]

**Supplementary Materials**

**Figure S1. E2F4 is a potential upstream regulator of AARS1 in HCC.**
(A) Venn diagram of candidate AARS1-regulating transcription factors predicted by hTFtarget, ChEA, GTRD, and ChIP-Atlas. (B) Correlation analysis between AARS1 and candidate transcription factors in the TCGA-LIHC cohort. (C) Positive correlation between E2F4 and AARS1 expression in TCGA-LIHC. (D) qRT-PCR analysis of E2F4 and AARS1 expression after E2F4 knockdown in Huh7 and SK-hep1 cells. (E) Western blot analysis of E2F4 and AARS1 protein levels after E2F4 knockdown. ACTIN served as the loading control.

Data are shown as mean ± SD; n = 3 independent experiments. The statistical significance was determined by Student’s two-tailed t test. *P < 0.05; **P < 0.01; ***P < 0.001; ***P < 0.0001.

**Figure S2. AARS1 promotes HCC progression in vitro and in vivo.**

**(A)** Western blot analysis of AARS1 expression in HepG2, LM3, Huh7, Hep3B, SK-hep1, and SNU449 cells. ACTIN served as the loading control. **(B, C)** Validation of AARS1 overexpression in Hep3B and SNU449 cells by RT–qPCR (B) and western blotting (C). Cells carrying the empty vector served as controls. **(D)** Cell proliferation assessed using CCK-8 assays at the indicated time points. **(E)** Representative images and quantification of colony formation in vector control and AARS1-overexpressing Hep3B and SNU449 cells. **(F)** Representative images of wound-healing assays and quantification of wound closure at 24 and 48 h. Red outlines indicate the wound boundaries. **(G)** Representative images and quantification of Transwell migration and invasion assays in the indicated cells. **(H)** Representative flow cytometry plots and quantification of early and late apoptosis assessed by Annexin V–APC/PI staining.

In vitro data are representative of three independent experiments. Quantitative data are presented as mean ± SD. Statistical analysis was performed using two-tailed Student’s t-tests or two-way ANOVA, as appropriate. ns, not significant (P > 0.05); *P < 0.05, **P < 0.01, ***P < 0.001, and ****P < 0.0001.

**Figure S3. AARS1 regulates global protein lactylation and promotes lactate production in HCC.**

(A)Representative immunofluorescence images showing lysine lactylation (Kla) staining in Huh7 and SK-hep1 cells (Scale bar, 10 μm.). (B)Correlation analysis between AARS1 expression level and lactate content in HCC specimens.

**Figure S4. Global landscape of the AARS1-regulated lactylome in HCC cells.**

(A) Differentially regulated proteins and modification sites in the siAARS1/NC comparison group.

**Figure S5. Identification of PHGDH as a target of AARS1-mediated lactylation.**
**(A)** Protein interaction network showing protein abundance changes following AARS1 knockdown. Node colors indicate log₂ fold changes (si_AARS1/NC), with PHGDH highlighted in red. **(B)** Venn diagram showing the overlap between potential AARS1-interacting proteins identified by anti-AARS1 immunoprecipitation followed by mass spectrometry (IP–MS) in Huh7 cells and potentially AARS1-regulated lactylated proteins identified by lactylomic analysis. **(C)** Representative tandem mass spectrometry (MS/MS) spectrum of the PHGDH-derived peptide AGTGVDNVDLEAATR, supporting PHGDH identification. Annotated b and y ions are shown in red and blue, respectively. **(D)** Immunoblot analysis of PHGDH lactylation in Huh7 and SK-hep1 cells expressing the indicated constructs following transduction with control shRNA (sh NC) or AARS1-targeting shRNA (sh AARS1). Cells were reconstituted with wild-type AARS1 (AARS1-WT) or AARS1-5A, which harbors five alanine substitutions (M46A/R77A/N216A/D239A/G241A) in the region associated with its lactyltransferase activity, together with Flag-PHGDH. Flag-PHGDH was immunoprecipitated using an anti-Flag antibody and immunoblotted for lysine lactylation (Kla) and Flag-PHGDH. Total cell lysates (TCL) were analyzed for AARS1, PHGDH, and ACTIN. **(E)** Immunoblot analysis of lysine acetylation (Kac) of immunoprecipitated Flag-tagged wild-type PHGDH or the K394R mutant in Huh7 and SK-hep1 cells. Whole-cell lysates (WCL) were analyzed for PHGDH and ACTIN. ACTIN served as a loading control. Blots in **(D, E)** are representative of three independent experiments.

**Figure S6. K394 lactylation of PHGDH enhances its enzymatic activity, promotes serine biosynthesis, and alleviates oxidative stress.**

(A)Flag-PHGDH-WT or Flag-PHGDH-K394R was transfected into PHGDH stable knockdown SK-hep1 cells. Flag-tagged PHGDH was immunoprecipitated using anti-Flag magnetic beads, followed by Western blot analysis to detect Kla and Flag signals. In a separate assay, PHGDH enzymatic activity was measured in whole-cell lysates using a colorimetric activity assay and normalized to the total protein concentration of each lysate. (B)PHGDH-stable knockdown SK-hep1 cells were reconstituted with Flag-PHGDH and transfected with siNC or siAARS1. Flag-PHGDH was immunoprecipitated and analyzed by western blot for Kla and Flag levels. In parallel, PHGDH activity was measured in whole-cell lysates using a colorimetric activity assay and normalized to the total protein concentration of each lysate. (C)PHGDH-stable knockdown SK-hep1 cells were reconstituted with Flag-PHGDH and transfected with Vector or AARS1. Flag-PHGDH was immunoprecipitated and analyzed for Kla and Flag by western blot. In parallel, PHGDH activity was measured in whole-cell lysates using a colorimetric activity assay and normalized to the total protein concentration of each lysate. (D)PHGDH-stable knockdown SK-hep1 cells were transfected with Flag-PHGDH-WT or Flag-PHGDH-K394R and treated with siNC or siAARS1. Flag-PHGDH was immunoprecipitated and analyzed for Kla and Flag by western blot. In parallel, PHGDH activity was measured in whole-cell lysates using a colorimetric activity assay and normalized to the total protein concentration of each lysate.

**Figure S7. PHGDH K394 contributes to AARS1-dependent malignant phenotypes in HCC cells, and β-alanine suppresses tumor growth.**
(A) Representative wound-healing images and quantification of wound closure at 24 and 48 h in SK-hep1 cells expressing control shRNA (sh NC) or PHGDH-targeting shRNA (sh PHGDH), together with empty vector, wild-type PHGDH, or the PHGDH K394R mutant, as indicated. (B) Representative images and quantification of Transwell migration and invasion assays in SK-hep1 cells with the same treatments as in (A). (C) CCK-8 assays assessing cell proliferation in PHGDH-depleted Huh7 and SK-hep1 cells reconstituted with wild-type PHGDH or the K394R mutant and transfected with control siRNA (si NC) or AARS1-targeting siRNA (si AARS1). (D, E) Representative images and quantification of colony formation (D) and Transwell migration (E) assays in Huh7 and SK-hep1 cells treated as described in (C). (F) Images of excised tumors, endpoint tumor weights, and tumor growth curves in nude mice bearing Huh7-derived xenografts in the control and β-alanine-treated groups (*n* = 5 mice per group). β-Alanine (Sangon Biotech, A606168) was supplied as a 1.2% (w/v) solution in drinking water, replaced weekly, and additionally administered by oral gavage at 200 μL per mouse three times per week using the same solution. Data in (A–E) are presented as the mean ± SD from three independent experiments. Statistical significance was assessed using two-tailed Student’s *t*-tests or two-way ANOVA, as appropriate. ***P* < 0.01; ****P* < 0.001; ns, not significant.
